# Enhanced peri-implantitis management through purple-LED irradiation coupled with silver ion application and calcium phosphate gene transfection carrier coating

**DOI:** 10.1038/s41598-025-96075-7

**Published:** 2025-04-21

**Authors:** Taito Iwabuchi, Taichi Tenkumo, Takayuki Mokudai, Masatoshi Takahashi, Toru Ogawa, Keiichi Sasaki, Nobuhiro Yoda

**Affiliations:** 1 Division of Advanced Prosthetic Dentistry, Tohoku University Graduate School of Dentistry, 4-1 Seiryo-Machi, Aoba-Ku, Sendai, 980-8575 Japan; 2 Joining and Welding Research Institute, Osaka University, 11-1 Mihogaoka, Ibaraki, 567-0047 Japan; 3 Institute for Materials Research, Tohoku University, 2-1-1 Katahira, Aoba-Ku, Sendai, 980-8577 Japan; 4 Division of Biomaterials and Bioengineering, School of Dentistry, Health Sciences University of Hokkaido, 1757 Kanazawa, Ishikari-Tobetsu, Hokkaido 061-0293 Japan; 5 Division of Comprehensive Dentistry, Tohoku University Graduate School of Dentistry, 4-1 Seiryo-Machi, Aoba-Ku, Sendai, 980-8575 Japan; 6 Tohoku University Graduate School of Dentistry, 4-1 Seiryo-Machi, Aoba-Ku, Sendai, 980-8575 Japan

**Keywords:** Silver, Purple LED, Hydroxyl radicals, Gene transfection, Calcium phosphate, Titanium surface, Peri-implantitis, Photocatalysis

## Abstract

The aim of this study was to investigate the bactericidal effect and recovery of biocompatibility of contaminated titanium surfaces using a combination treatment involving silver, copper, or iron ion application along with 400 nm purple-LED light irradiation. Additionally, the study sought to develop a functional calcium phosphate (CaP) coating treatment on titanium surfaces following disinfection, to promote re-osseointegration. A purple-LED emitting light at 400 nm was utilized to irradiate *Staphylococcus aureus* suspensions and biofilms in the presence of various concentrations of silver, copper, and iron solutions for 1 min. The bactericidal effect and electron spin resonance (ESR) spectrum were subsequently evaluated. Additionally, the hydrophilicity of the titanium surface and cell viability of MC3T3-E1 cells after combination treatment with silver ion was evaluated. Furthermore, a titanium surface coating with CaP gene transfection carrier containing plasmid DNA was developed using an electric current. The activity of hard tissue formation was then evaluated both in vitro and in vivo post-treatment. The bactericidal effect of the combination treatment with silver ions was attributed to the generation of hydroxyl radicals, whereas the effects from iron and copper treatments were not radical-mediated. The silver treatment significantly restored the hydrophilicity and cell affinity of the titanium surface. Moreover, CaP coating applied via an electric current (30 µA for 5 min) enhanced hard tissue formation activity on the titanium surface in both in vitro and in vivo settings. The combination treatment utilizing silver ions and purple-LED irradiation significantly enhanced bactericidal effects by generating high levels of hydroxyl radicals. Additionally, coating the titanium surface with functionalized CaP promoted early osseointegration, suggesting a promising strategy for improving implant outcomes.

## Introduction

With the global proliferation of implant treatments^1^, an increase in the incidence of peri-implantitis has been documented^2,3^. Various conventional devices and chemical agents utilized for periodontal treatment^4^, including laser therapy^5–7^, ultrasonic scalers^8^, electrolytic Cleaning^9^, and air abrasion^7^, have been employed to manage peri-implantitis.

Photodynamic therapy (PDT) has recently garnered attention as an innovative therapeutic approach for peri-implantitis^10^. PDT involves administering a photosensitizing agent and subsequently irradiating the target tissue with light of a specific wavelength, which induces the production of reactive oxygen species from the photosensitizing agent. This technique is widely utilized in the fields of neurosurgery and dermatology, and has been introduced as a novel treatment modality for peri-implantitis in dentistry^11^. Nevertheless, biofilms on microstructured titanium surfaces are challenging to eliminate and obstruct bone reattachment by hindering protein and blood adhesion to the titanium surface post-decontamination^12^. It has been reported that ultraviolet C (UVC) irradiation of aged titanium surfaces restores hydrophilicity through photocatalytic activity, thereby facilitating osseointegration^13,14^. However, the application of UVC in the oral cavity presents difficulties due to its extremely short wavelength (200–280 nm)^15^. Tenkumo et al. demonstrated that UVA (365 nm) irradiation of bacteria in the presence of silver ions generates hydroxyl radicals, enhancing the bactericidal effect^16^. Nakamura et al. further noted that hydroxyl radicals can be generated by LED irradiation at 400 nm using 4 mmol/L gallic acid^17^.

Hydroxyl radicals are recognized not only for their bactericidal properties but also for their ability to enhance the titanium surface area^14,18^. Additionally, metal ions such as copper and iron are recognized to act as cluster catalysts, producing photocatalytic activity in the visible-light spectrum^19,20^. In contrast, silver ions possess intrinsic antimicrobial properties^21^, with silver diamine fluoride and other silver compounds utilized to prevent dental caries^22^. Furthermore, iron and copper ions exhibit bactericidal activities^19,23^. Based on these findings, visible-light irradiation treatment following the application of these metal ions to a contaminated titanium surface may enhance disinfection and restore the biocompatibility of the titanium surface via photocatalytic mechanisms.

In periodontal surgery involving implants, epithelial or connective tissue-derived cells are the first to adhere to the surfaces during the healing process following sterilization^24^. Epithelial attachment, connective tissue attachment, and gingival recession have been observed on titanium surfaces post-sterilization. Therefore, it is crucial to provide titanium surfaces with functionalities that promote bone tissue reattachment post-sterilization. Titanium surfaces modified with N-halamine polymers, tantalum, and silver nanoparticles demonstrate superior osseointegration compared to conventional titanium surfaces^25–27^. However, existing studies have predominantly focused on coating implants prior to placement, and there is a lack of research regarding the coating of implants already positioned in the body that are impacted by peri-implantitis. Thus, a technique for post-placement coating of titanium surfaces is necessary to promote re-osseointegration.

In our previous investigation, we induced bone tissue formation using functional calcium phosphate nanoparticles (CaP) as a carrier for plasmid DNA, facilitating gene transfection^28^. Cells that internalize CaP loaded with plasmid DNA can release targeted proteins or cytokines, resulting in either bone formation or the inhibition of inflammation ^29,30^. The CaP nanoparticles are positively charged^29,30^, while the cell or titanium surface is negatively charged^31^, promoting interactions via electrostatic forces. The coating of CaP with plasmid DNA containing Bone Morphogenetic Protein-2 (BMP-2) on the surfaces of titanium implants can promote re-osseointegration.

The proposed treatment method, involving the application of metal ions alongside irradiation with 400 nm visible light on titanium surfaces affected by peri-implantitis, as well as subsequent functional CaP coating, represents a comprehensive approach to addressing the aforementioned issues. This method is expected to promote osseointegration through the bactericidal effects of hydroxyl radicals on biofilms and the induction of hard tissue formation via gene delivery to the cells adhered to the titanium surface. Therefore, in this study, we evaluated the effectiveness of this hypothesis as follows:

1. To detect the bactericidal effect and underlying mechanism: X-band electron spin resonance (ESR) spectra of the reacted solution and antibacterial tests were conducted 48 h post-400 nm purple LED light irradiation in the presence of silver, copper, and iron ions against *Staphylococcus aureus* suspension or biofilms formed on the titanium surface.
2. To assess the biocompatibility of the titanium surface post-treatment: The hydrophilic properties and MTT assay for osteoblast-like cells (MC3T3E1) were conducted following purple LED irradiation on biofilms and application of these metal ions.
3. To evaluate the coating methodology for functional CaP: The volume of CaP attached to titanium surfaces under various conditions was quantified using inductively coupled plasma optical emission spectrometry (ICP-OES).
4. To investigate the hard tissue formation activity of the CaP coating on titanium surfaces: Alkaline phosphatase (ALP) activity assays or calcification tests in vitro and removal torque tests of implanted CaP-coated titanium screws were performed.

## Results

### Bactericidal effect and radical generation

**Fig. 1:**
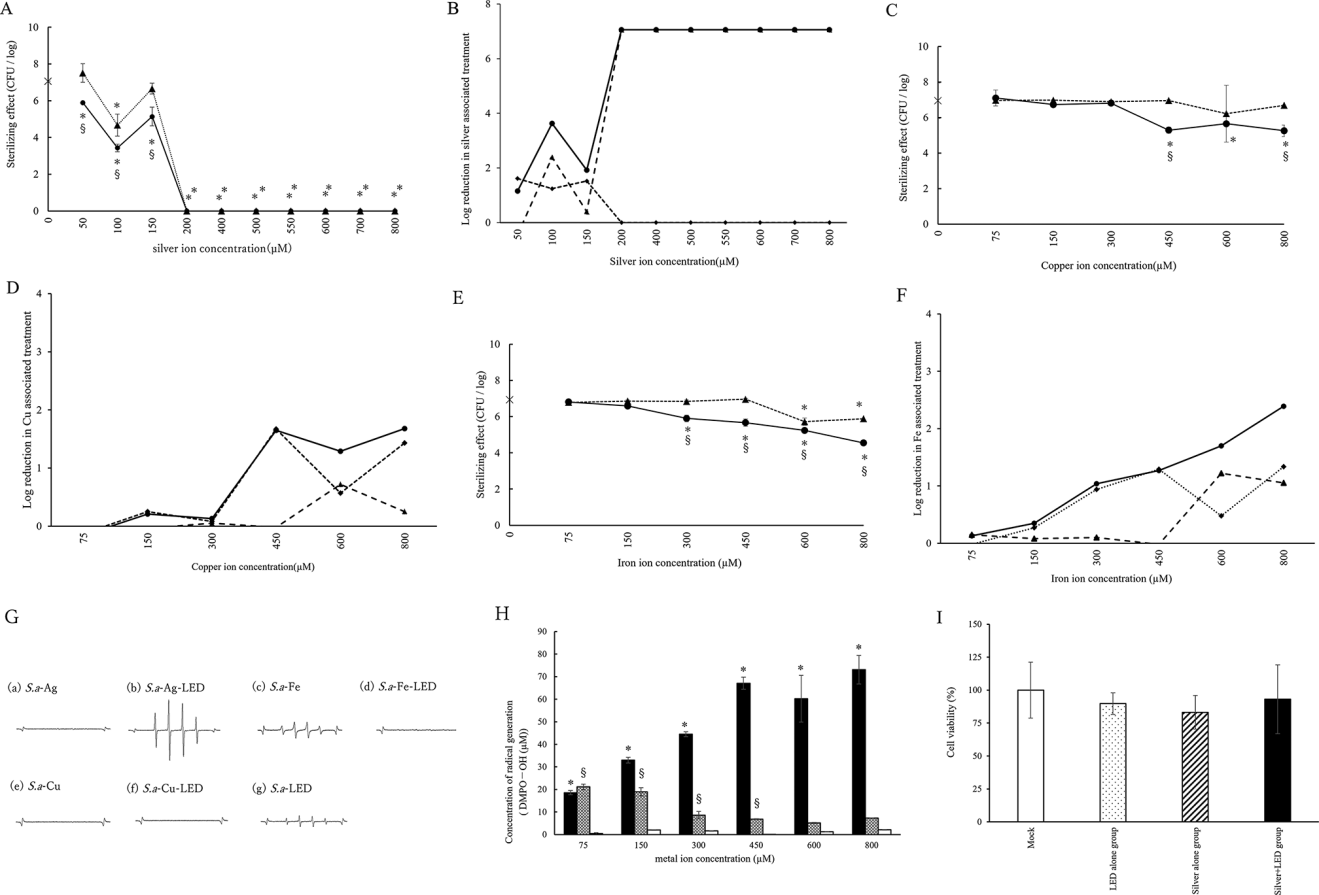
(**A**) Bactericidal effect of Ag-purple LED treatment on suspended bacteria. Symbol ●, ▲, and × demonstrate Silver + LED treatment, Silver − No LED treatment, and Control (water) − No LED groups, respectively. *Significant differences compared to Control (water)-No LED group (p < 0.05). ^§^Significant differences between Silver + LED treatment and Silver-No LED treatment groups (p < 0.05). (**B**) Log reduction (●) between Silver + LED treatment group and Control (water)-No LED groups, (▲) Silver-No LED treatment group and Control (water)-No LED groups, (◆) Silver + LED treatment group and Silver-No LED treatment group. (**C**) Bactericidal effect of Cu-purple LED treatment on suspended bacteria Symbols ●, ▲, and × demonstrate Copper + LED treatment, Copper-No LED treatment, and Control (water)-No LED groups, respectively. *Significant differences compared to Control (water)-No LED group (p < 0.05). ^§^Significant differences between Copper + LED treatment and Copper-No LED treatment groups (p < 0.05). (**D**) Log reduction (●) between Copper + LED treatment group and Control (water)-No LED groups, (▲) Copper-No LED treatment group and Control (water)-No LED groups, (◆) Copper + LED treatment group and Copper-No LED treatment group. (**E**) Bactericidal effect of Fe-purple LED treatment on suspended bacteria Symbols ●, ▲, and × demonstrate Iron + LED treatment, Iron-No LED treatment, and Iron-No LED treatment groups, respectively. *Significant differences compared to Control (water)-No LED group (p < 0.05). ^§^Significant differences between Iron + LED treatment and Iron-No LED treatment groups (p < 0.05). (**F**) Log reduction (●) between Iron + LED treatment group and Control (water)-No LED groups, (▲) Iron-No LED treatment group and Control (water)-No LED groups, (◆) Iron + LED treatment group and Iron-No LED treatment group. (**G**) Representative electron spin resistance (ESR) spectra of the radicals generated by (a) *S. a*-Ag, (b) *S. a*-Ag-LED, (c) *S. a*-Fe, (d) *S. a*-Fe-LED, (e) *S. a*-Cu, (f) *S. a*-Cu-LED, and (g) *S. a*-LED. (**H**) Radical generation at metal ion concentrations Symbols ■, ▩, and □ demonstrate Silver + LED treatment, Copper + LED treatment, and Iron + LED treatment groups, respectively. *Significant differences between Silver + LED treatment and Copper + LED treatment groups (p < 0.05). ^§^Significant differences between Iron + LED treatment and Copper + LED treatment groups (p < 0.05). (**I**) Comparison of cell viability of the combination treatment (Silver + LED), silver ion application (Silver alone), or purple LED irradiation (LED alone). No significant differences were noted among all groups.

The bactericidal effects of silver ions on floating *Staphylococcus aureus* are shown in Fig. 1A. The Silver + LED treatment group showed significantly higher bactericidal effect compared to the Control (water)-No LED group at all concentrations, and Silver-No LED treatment group at 50, 100, and 150 µM concentrations. The Silver-No LED treatment group showed significantly higher bactericidal efficacy than the Control (water)-No LED group at 100 µM and over 200 µM. A reduction exceeding 3 logs compared to the Control (water)-No LED group was observed in the Silver + LED treatment group at concentrations exceeding 100 μM, while a reduction over 200 μM was noted in the Silver-No LED treatment group. However, a reduction exceeding 3 logs between the Silver + LED treatment and Silver-No LED treatment groups was not observed (Fig. 1B).

Regarding copper ions, the Copper + LED treatment group showed significantly higher bactericidal effect than the Control (water)-No LED group at a concentration of over 450 µM, and the Copper-No LED treatment group at concentrations of 450 and 800 µM (Fig. 1C). No significant differences were found between the Copper-No LED treatment and Control (water)-No LED groups. In the Copper + LED treatment and Copper-No LED treatment groups, reductions exceeding 3 logs compared to the Control (water)-No LED group were not observed (Fig. 1D).

Regarding iron ions, the Iron + LED treatment group showed significantly higher bactericidal effect than Iron-No LED treatment and Control (water)-No LED treatment groups at concentrations exceeding 300 µM (Fig. 1E). The Iron-No LED treatment group showed significantly higher bactericidal effect than the Iron-No LED treatment group at a concentration of over 600 µM. No reductions exceeding 3 logs compared to the Control (water)-No LED group were noted in either the Iron + LED treatment or Iron-No LED treatment groups (Fig. 1F).

Representative electron spin resistance (ESR) spectra of the radicals generated by the Silver + LED treatment, Copper + LED treatment, and Iron + LED treatment are shown in Fig. 1G. The presence of DMPO-OH was confirmed by a hyperfine coupling constant (hfcc) of aN = aH = 14.9 G. The S.a-Ag-LED group showed a clear waveform compared to S.a-Ag group. The ESR spectra of S.a-Ag-LED resembled those of hydroxyl radicals. Conversely, the S.a-Fe group showed clear waveform compared to the S.a-Fe-LED group. The ESR spectra of S.a-Fe were identical to those of thiol radicals reported in previous research^32^. Both S.a-Cu group and S.a-Cu-LED group showed no clear waveform. S.a-LED group showed small waveform. The amounts of radicals generated by the Silver + LED treatment, Copper + LED treatment, and Iron + LED treatment are shown in Fig. 1H. The number of radicals generated with Silver + LED treatment increased with increasing silver concentrations and was significantly higher than that generated by Copper + LED treatment and Iron + LED treatments at all concentrations. No significant differences were noted for number of radicals generated with silver + LED treatment at concentrations of 450 μM or higher. The number of radicals generated with Iron + LED treatment decreased with increasing iron concentrations, whereas that generated with Copper + LED treatment did not depend on copper concentration. The number of radicals generated with Iron + LED treatment at concentrations of 75 to 450 μM was significantly higher than that with Copper + LED treatment at same concentrations.

The results of cell cytotoxicity across treatment groups are depicted in Fig. 1I. No significant differences were observed among all groups (Mock, purple-LED irradiation alone, silver application alone, and silver + LED).

**Fig. 2:**
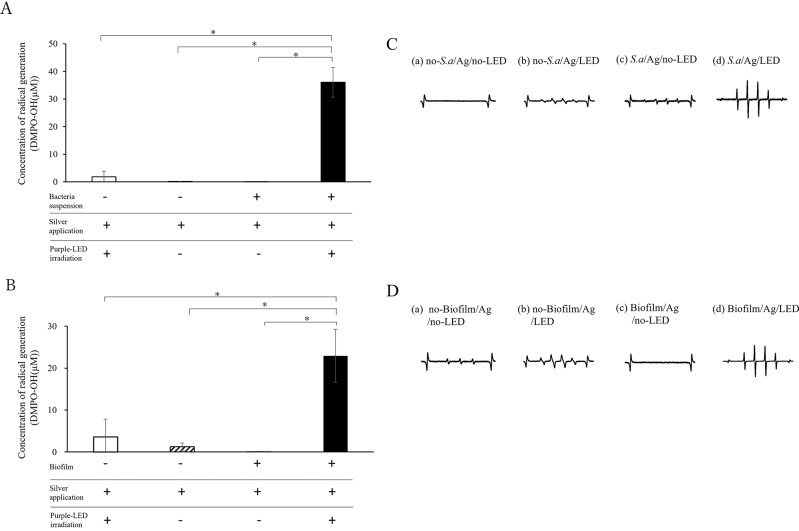
(**A**) Generated radicals in silver ion solution with and without bacterial suspension and purple LED irradiation. *Significant differences (p < 0.05). (**B**) Radicals generated in silver ion solution with and without biofilms on titanium disks, under purple LED irradiation. *Significant difference (p < 0.05). + indicates application or irradiation. (**C**) Representative electron spin resistance (ESR) spectra of the radicals generated by the following treatments: (a) bacterial suspension(−) silver application(+) Purple-LED irradiation(−), (b) bacterial suspension(−) silver application(+) Purple-LED irradiation(+), (c) bacterial suspension(+) silver application(+) Purple-LED irradiation(−), and (d) bacterial suspension(+) silver application(+) Purple-LED irradiation(+). (**D**) Representative ESR spectra of the radical generated by the following treatments: (a) Titanium with biofilm(−) silver application(+) Purple-LED irradiation(−), (b) Titanium with biofilm(−) silver application(+) Purple-LED irradiation(+), (c) Titanium with biofilm(+) silver application(+) Purple-LED irradiation(−), and (d) Titanium with biofilm(+) silver application(+) Purple-LED irradiation(+).

The quantity of DMPO-OH generated from Ag-purple LED irradiation of the bacterial suspension was significantly higher across all groups (Fig. 2A). Waveforms indicative of hydroxyl radicals were detected after purple LED irradiation of a mixture comprising bacterial suspension and silver solution (Fig. 2C). Radical generation was observed following the combination with Ag-purple LED irradiation treatment or the mixture of silver ions and bacterial suspensions.

Consistent with the bacterial suspension, the amount of DMPO-OH generated from purple Ag LED irradiation of the biofilm on the titanium surface was significantly higher across all groups (Fig. 2B). Waveforms suggestive of hydroxyl radicals were again observed after purple LED irradiation of a mixture containing bacterial suspension and silver solution (Fig. 2D). The intensity of radicals generated through the combined treatment of Ag-purple LED irradiation on the titanium surface exceeded that of silver application alone.

### Titanium surface properties

The charge on a titanium surface without current application was − 1.88 C, while that of the titanium surface upon application of a current of 10 mA and a voltage of 0.5 V disk was − 31.62 C.

**Fig. 3:**
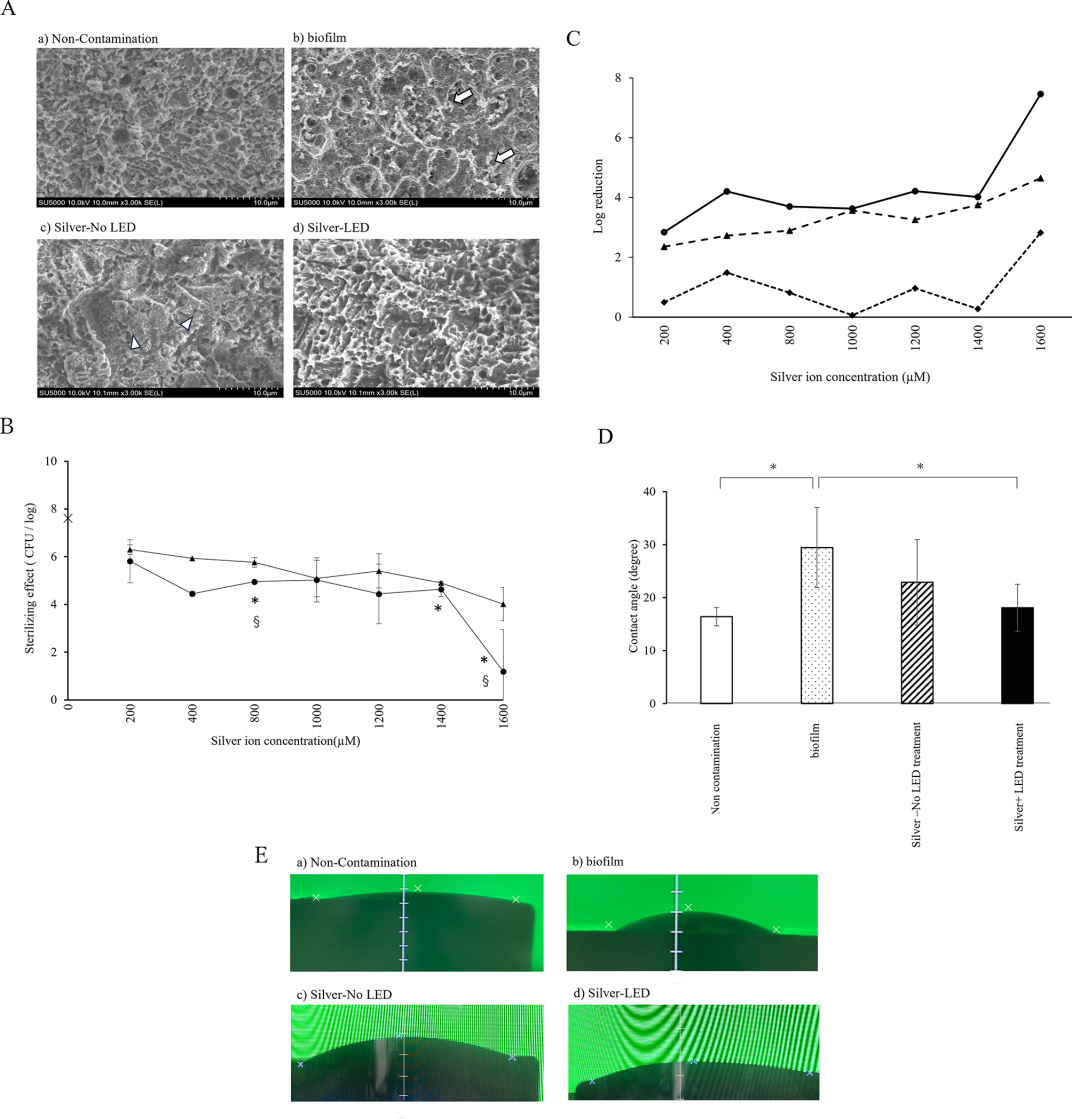
(**A**) Scanning electron microscopy (SEM) images of titanium surfaces in a) non-contamination group, b) biofilm, c) Silver-No LED treatment group, and d) Silver-LED treatment group. Symbols ⇒ and △ shows bacteria and granule deposits, respectively. (**B**) Bactericidal effects of silver ion on biofilm on titanium. Symbols ●, ▲, and × demonstrate Silver + LED treatment, Silver-No LED treatment, and Control (water)-No LED groups, respectively. *Significant differences between Silver + LED treatment and Control (water)-No LED groups (p < 0.05). ^§^Significant differences between Silver + LED treatment and Silver-No LED treatment groups (p < 0.05). (**C**) Log reduction (●) between Silver + LED treatment group and Control (water)-No LED groups, (▲) Silver-No LED treatment group and Control (water)-No LED groups, (◆) Silver + LED treatment group and Silver-No LED treatment group. (**D**) Evaluation of contact angle in non-contamination, bacteria-attached group, Silver-No LED treatment group, and Silver-No LED treatment group. * Significant difference (P < 0.05). (**E**) An example of contact angle of titanium disk in a) Non contamination group: 17.4°, b) Biofilm group: 40.1°, c) Silver-No LED treatment group: 22.4°, and d) Control (water)-No LED group: 21.9°.

SEM images depicting the titanium surface before and after the application of silver and/or purple LED irradiation of biofilms are shown in Fig. 3A. The titanium surface in the non-contamination group exhibited roughness, and bacteria were identified on the rough surface within the biofilm group. In the Silver-No LED treatment group, no apparent bacteria were observed on the titanium surface; however, fine granules were detected. Neither bacteria nor granules were observed in the Silver + LED treatment group. The bactericidal effects of silver ions on the biofilm of *Staphylococcus aureus* with and without UV-A irradiation are shown in Fig. 3B. The Silver + LED treatment group indicated significantly greater bactericidal effects compared to the biofilm group at concentrations of 800, 1400, and 1600 µM. A reduction exceeding 3 CFU/log compared to the biofilm group was noted over 400 μM in the Silver + LED treatment group, while reductions were observed over 1000 μM in the Silver-No LED treatment group (Fig. 3C). The reduction of over 3 log CFU/log between the Silver + LED treatment and Silver-No LED treatment groups was not recorded.

In terms of the hydrophilicity of the titanium surface, the contact angles of the biofilm groups were significantly larger than those of the non-contamination and Silver + LED treatment groups. There was no significant difference in the contact angle between non-contamination, Silver-No LED treatment, and Silver + LED treatment groups (Fig. 3D, E).

### Cell viability on titanium surface after treatment

**Fig. 4:**
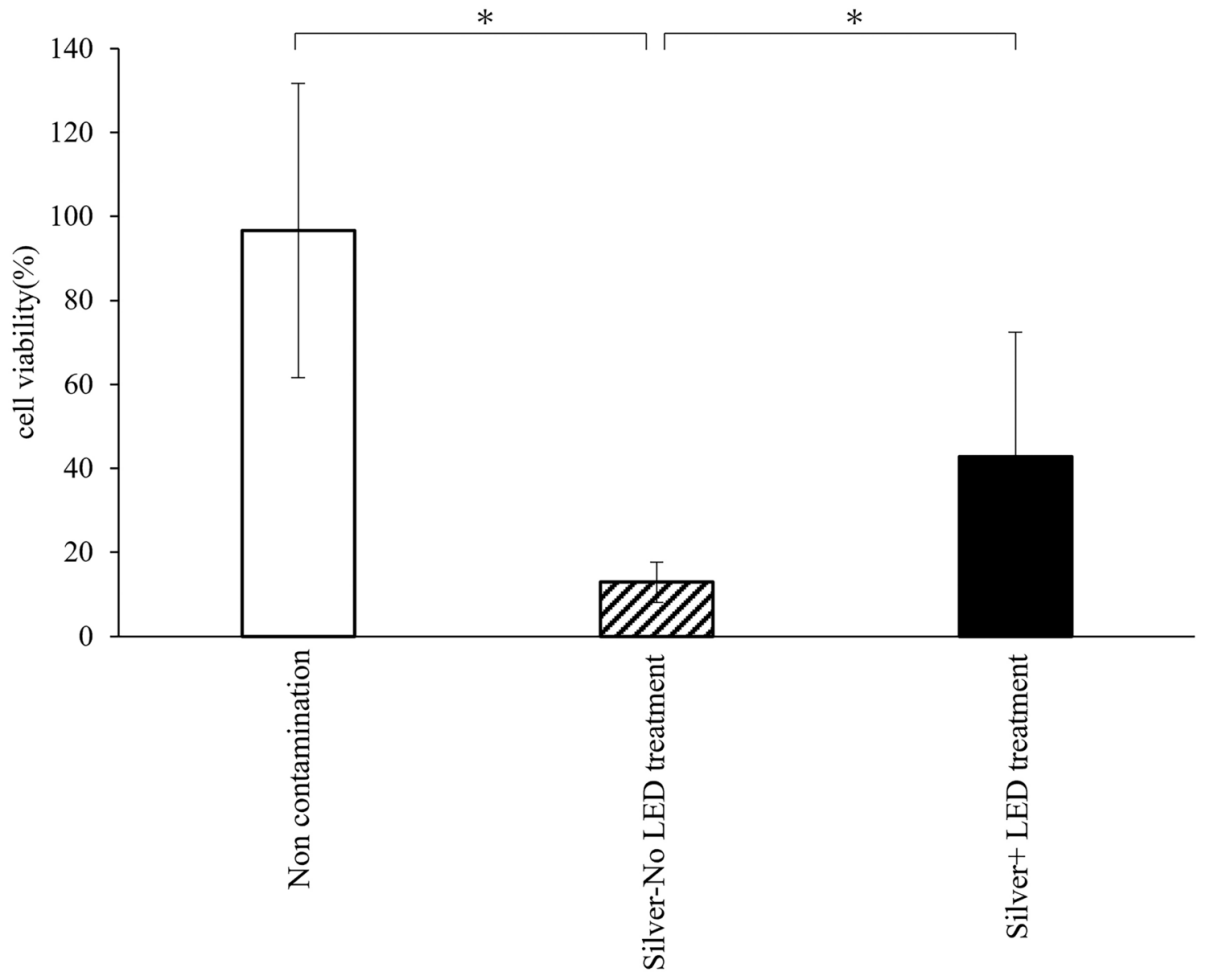
Comparison of cell viability under conditions of silver application and/or LED treatment. *Significant difference (p < 0.05).

Cell viability on titanium surfaces following Silver-No LED treatment or Silver + LED treatment is depicted in Fig. 4. The cell viability within the non-contamination and Silver + LED treatment groups was significantly higher than that of the Silver-No LED treatment group.

### CaP coating on titanium surface

**Fig. 5:**
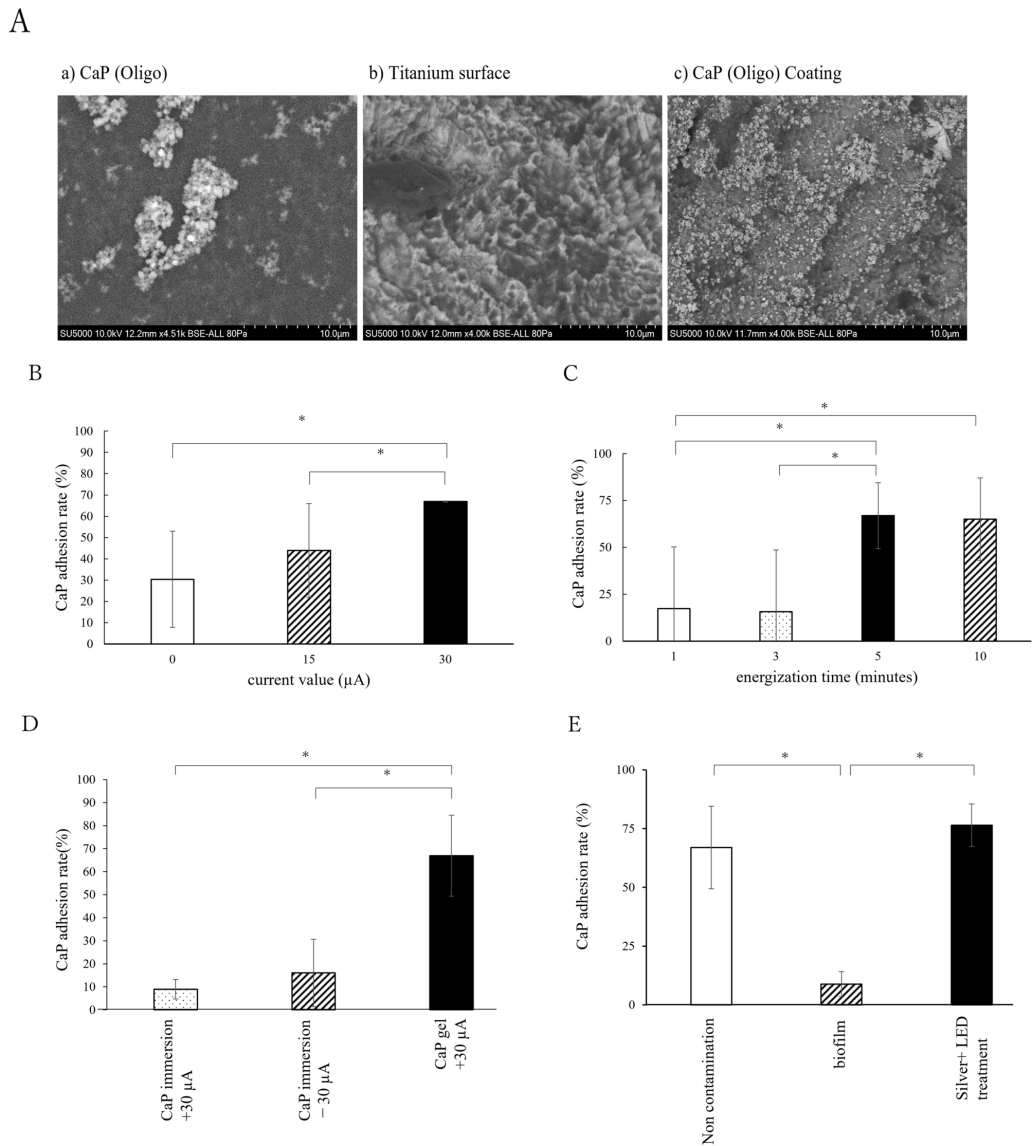
(**A**) Scanning electron microscopy (SEM) images of a) CaP(oligo), b) titanium surface c) CaP(oligo) coating on titanium surface. (**B**) CaP adhesion rate on titanium surface based on different current values. (**C**) CaP adhesion rate on titanium surface based on different energization times at 30 μA. (**D**) CaP adhesion rate on titanium surface based on different coating conditions. (**E**) Comparison of CaP adhesion rates under different titanium surface conditions with and without Ag-purple LED treatment. *Significant difference (p < 0.05); *CaP* calcium phosphate nanoparticles.

The size, zeta potential, and dielectric constant of CaP nanoparticle were 114 ± 19 nm, + 10.7, and 78.3, respectively. The conductivity of the CaP suspension was 1832.5 μs/cm. SEM images of CaP revealed granules of approximately 300 µM in diameter (Fig. 5A).

### CaP coating method

When maintaining a constant duration of current application (5 min), the adhesion of CaP was significantly higher at a current of 30 μA compared to conditions of 0 and 15 μA (Fig. 5B). Conversely, when the current was set at 30 μA, significantly higher CaP adhesion was observed at 5 min and 10 min (Fig. 5C); however, no statistically significant difference was noted between the 5-min and 10-min durations. Regarding the CaP coating method, the application of current directly to a titanium disk coated with a CaP gel (CaP gel + 30 μA) yielded a greater amount of CaP adhesion compared to immersing the titanium disk in a CaP solution and applying current (CaP immersion + 30 μA and CaP immersion -30 μA). When immersing the titanium disk in a CaP solution with current application, no statistical difference in CaP adhesion was observed between the anode and cathode sides of the disk (Fig. 5D). Significantly greater CaP adhesion was detected in the Silver + LED treatment group than in the biofilm group, and it was comparable to the non-contaminated group (Fig. 5E). Spattered nanoparticles were identified on the titanium surface (Fig. 5A).

### Functional evaluation of CaP coating

**Fig. 6:**
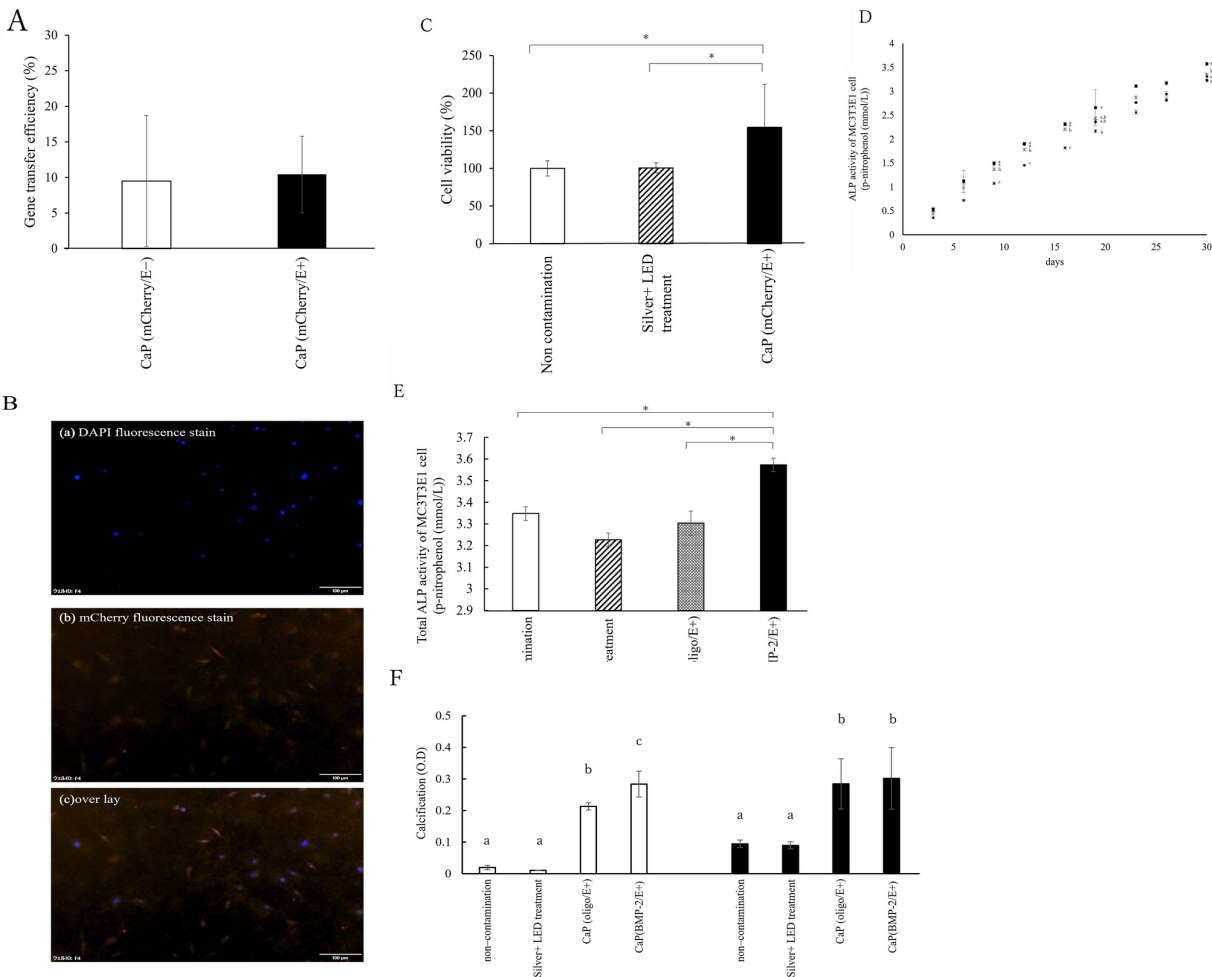
(**A**) Gene transfection efficiency of CaP loaded with plasmid DNA encoding mCherry with CaP (mCherry/E+) and without CaP (mCherry/E−), electric current set to 30 μA. (**B**) Example images of a) DAPI fluorescence stain, b) mCherry fluorescence stain, and c) overlay of the gene transfection efficiency of CaP on the titanium surface after Silver + LED treatment. (**C**) Viability of MC3T3E1 cells on treated titanium surface after 3 days of application with CaP (mCherry) with electronic current set at 30 μA. *Significant difference (p < 0.05). (**D**) ALP activity of MC3T3E1 cells after the application of CaP (Oligo) or CaP(BMP-2) with electronic current at 30 μA. Symbols ×, ●, ◆, and ■□ represent non-contamination, Silver + LED treatment, CaP(oligo/E+), and CaP(BMP-2/E+), respectively. Significant differences (p < 0.05) between groups are denoted by different superscript letters (the same letter indicates no significant difference). (**E**) Total ALP activity of MC3T3E1 cells after 30 days the application of CaP (Oligo) or CaP (BMP-2) with electronic current at 30 μA. *Significant difference (p < 0.05). (**F**) Cell calcification on the titanium surface treated with Silver + LED treatment followed by the application of CaP (Oligo) or CaP (BMP-2) with electronic current set at 30 μA in MC3T3E1 cells at 14 (□) or 28 days (■□). Significant differences (p < 0.05) between groups are denoted by different superscript letters (the same letter is not significantly different).

The gene transfection efficiency of CaP (mCherry/E+) did not significantly differ from that of CaP (mCherry/E-) (Fig. 6A,B). In term of the ability of the CaP coating to sustain cell viability, the CaP (oligo/E+) group exhibited significantly higher cell viability compared to non-contamination and Silver + LED treatment groups (Fig. 6C). No significant difference in cell viability was noted between the non-contamination and Silver + LED treatment groups.

ALP activity increased over time in all groups (Fig. 6D). The CaP(BMP-2/E+) group displayed significantly higher total ALP activity at 30 days post-treatment compared to the other groups (Fig. 6E).

In the calcification test, the CaP (BMP-2/E+) group showed significantly greater values at 14 days post-treatment than all other groups, and significantly higher values at 28 days post-treatment compared to both the non-contaminated and Silver + LED treatment groups (Fig. 6F).

**Fig. 7:**
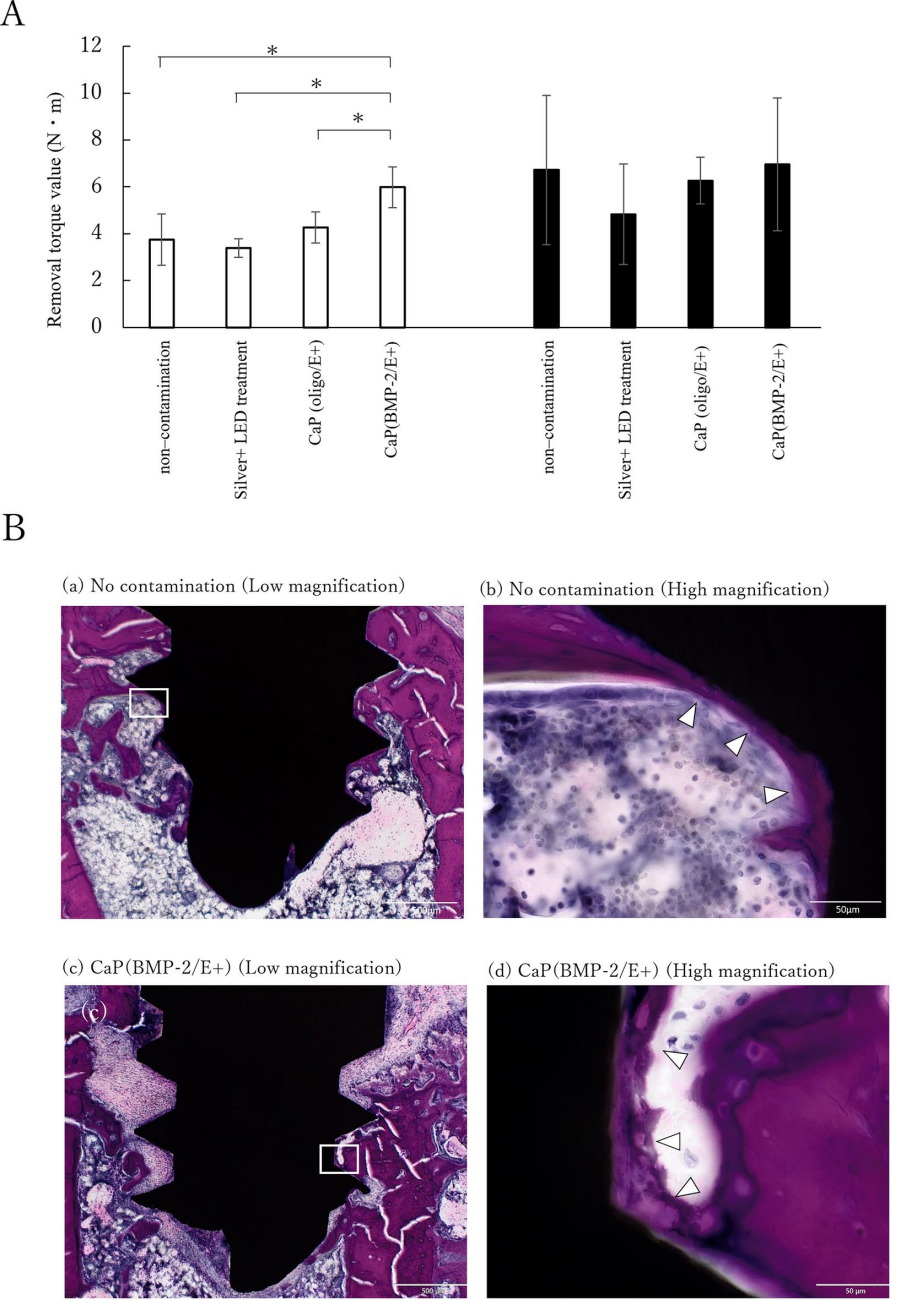
(**A**) Removal torque test in rat tibia 14 days (□) and 28 days (■□) post-implantation. *denotes significant differences (p < 0.05). (**B**) Histological images of the tissue surrounding implanted titanium screws 28 days after implantation in the rat tibia. Histological assessment of the non-contamination titanium screw (a,b) and titanium screws in the CaP(BMP-2/E +) group (c,d) after staining with hematoxylin and eosin (H–E). Scale bars: 500 μm (a, c) and 50 μm (b, d). H&E-stained histology around the titanium screws 28 days post-implantation in rat femurs. △ shows direct bone attachment on the implant screw surface.

In the in vivo experiments, the removal torque of the CaP (BMP-2/E+) groups was significantly higher at 14 days post-treatment compared to all other groups, whereas no significant differences were observed at 28 days post-treatment among all groups (Fig. 7A). Direct bone attachment to the implant surface was observed in both non-contamination and CaP (BMP-2/E+) groups (Fig. 7B).

## Discussion

In this study, we aimed to investigate the bactericidal effects and the recovery of biocompatibility of contaminated titanium surfaces through a combination treatment involving silver, copper, or iron ion application alongside purple-LED light irradiation at 400 nm. Additionally, we sought to develop a functional CaP coating treatment on titanium surfaces following disinfection to promote re-osseointegration. Our findings demonstrated that the combination treatment effectively enhanced bactericidal activity against *Staphylococcus aureus,* with the silver ion treatment generating hydroxyl radicals, while treatments with iron and copper did not rely on radical formation. Furthermore, the application of silver ions not only restored the hydrophilicity and cell affinity of the titanium surface but also showed promising results in terms of hard tissue formation activity when coupled with CaP coating using electrical current.

Our study has the potential to advance treatment strategies for peri-implantitis and enhance the biocompatibility of titanium implants.

### Bactericidal effect and recovery of the titanium surface after treatment with silver Ions and purple-LED irradiation

First, we investigated whether iron, copper, and silver ions enhance the bactericidal effects under light irradiation at a wavelength of 400 nm. In dentistry, light with wavelengths ranging from 380 to 420 nm and 420–495 nm is commonly utilized in light-curing equipment^33^. Typically, shorter wavelengths exhibit higher energies and greater biological effects. Visible light at 400 nm is considered to have lower energy and potentially milder biological impacts compared to 365 nm of UV-A; hence, this study employed a purple LED with a wavelength of 400 nm. Furthermore, the antibacterial effect of silver persisted as long as contact with the bacteria was maintained. To mitigate excessive reactions, the bactericidal effect was assessed by adding a MHB solution to the reaction mixture, as previously described by Tenkumo et al.^16^.

The U.S. Environmental Protection Agency Office of Pesticide Programs (EPA) require a minimum reduction of 3 logs in test organisms within 1–2 h as the requisite performance level for evaluating antimicrobial surface coatings. Silver ion application alone showed a bactericidal effect with reductions exceeding 3 logs in CFU, over 200 µM, whereas free radicals were not generated. This indicated that the bactericidal effect of silver ions was not radical-mediated. In contrast, the combined treatment with purple LED irradiation at 400 nm and silver ions exhibited an amplified bactericidal effect over 50 µM while generating hydroxyl radicals. Tenkumo et al. reported that the inclusion of a hydroxyl radical scavenger diminished the bactericidal effect of a combination treatment of silver and light irradiation at 365 nm^16^. The hydroxyl radicals generated in this combination with silver may have augmented the bactericidal effect observed in this study. An increase in the bactericidal efficacy of the combined treatment of silver application and purple LED irradiation was noted in a silver concentration-dependent manner, though a reduction was observed at 150 μM. This trend has also been reported by Tenkumo et al. in studies using *A. actinomycetemcomitans* and *S. mutans*^16^. Although an appropriate correlation between bacterial count and silver concentration may exist, the underlying cause of this phenomenon was not elucidated in this study.

However, bactericidal effect of the copper combined with purple LED treatment was found to be superior compared to the treatment without purple LED irradiation; however, no hydroxyl radicals were detected in the combination treatment. Iron ions exhibited a concentration-dependent increase in bactericidal effect, whereas the quantity of radicals generated exhibited a concentration-dependent reduction. Despite the absence of radical generation, the combination treatment involving high concentrations of iron or copper ions demonstrated a greater bactericidal effect than that observed with iron or copper ions alone. This indicates that visible light at a wavelength of 400 nm may exert a bactericidal effect. However, reductions exceeding 3 logs in CFU compared to untreated controls were not observed with purple LED irradiation applied to copper or iron, while the bactericidal effect of the suspension was investigated in this study. The clear bactericidal effect of copper or iron, regardless of purple LED irradiation, was not observed under the described study conditions. This suggests that the synergy of purple LED irradiation and the application of iron or copper kills bacteria via mechanisms distinct from radical-mediated processes. Although the bactericidal mechanism of copper ions remains enigmatic, existing literature suggests that it may involve a sequence of membrane damage, copper influx into cells, oxidative damage, and consequent DNA degradation^19,34,35^. The bactericidal effect of iron ions is attributed to the damage to the bacterial cell surface layer by divalent iron and the free radical reaction mechanisms occurring intracellularly^36^. In this study, radicals were only generated by mixing iron ions with cell suspensions, while purple LED irradiation served to inhibit this process. The Fenton effect occurs when hydrogen peroxide (H₂O₂) interacts with divalent iron ions (Fe^2^⁺) to generate hydroxyl radicals (-OH) and trivalent iron ions (Fe^3^⁺); iron nitrate, a trivalent ion, was utilized in this study. The reduction of trivalent iron ions to divalent iron ions within bacteria via irradiation could potentially trigger the Fenton reaction. However, the combination of trivalent iron ions and purple LED irradiation may impede radical generation in the Fenton reaction.

Comparatively, greater concentrations of silver nitrate were necessary to demonstrate bactericidal effects on titanium disks than on suspended bacteria. This discrepancy may stem from the protective nature of biofilms against silver and light irradiation^37^. Ogawa et al. reported that UVC irradiation of aged titanium surfaces restored hydrophilicity of the titanium surface due to photocatalytic activity^14^. In this study, the combined Ag-purple LED treatment effectively decreased the contact angle, indicating restoration of titanium surface hydrophilicity, potentially hypothesizing the role of silver ions and bacteria as co-catalysts for the photocatalytic effects elicited by the purple LED.

The intensity of radicals generated through the combined treatment of Ag-purple LED irradiation on the titanium surface was found to be greater than that of silver application alone. Zhang et al. determined that silver ions function as co-catalysts on titanium surfaces^38^. Tenkumo et al. highlighted that UVA (365 nm) irradiation alongside silver ions restored cellular viability^16^. Nakamura et al. indicated that UVA irradiation of hydrogen peroxide generates hydroxyl radicals, enhancing titanium surface hydrophilicity and, consequently, cellular viability^39^. Collectively, these findings suggest that visible light at a wavelength of 400 nm, in conjunction with silver application, improved titanium surface hydrophilicity and restored cellular viability. Conversely, hydroxyl radicals not only possess bactericidal properties but also exhibit cytotoxic effects on cells^40^. However, no significant cell cytotoxicity from the combined treatment was observed in this study. The volume of hydroxyl radicals generated in this investigation might not have impacted cell proliferation due to its minimal quantity. Tenkumo et al. reported that the toxicity of the combination treatment involving silver and UVA irradiation on cell proliferation was not significantly different from that of silver application alone^16^. However, the effects of the combination treatment on cell function or DNA damage remain unclear; thus, further investigation into the influence of the combination treatment on cellular activity is warranted in future research.

### Electrical adhesion of functionalized CaP on titanium surface

Numerous studies have developed CaP-like hydroxyapatite coatings on titanium implant surfaces demonstrating augmented osseointegration associated with CaP coatings^41^; however, these interventions are predominantly ex vivo and physically unattainable for implants once placed in the body. Therefore, a CaP coating method that can be applied in vivo was developed in this study.

The surface charge of the titanium disk was negative, and this negative charge further intensified upon the application of an electric current. Wu et al. reported a titanium surface zeta potential of − 33.86^31^; however, the zeta potential of CaP was reported to be positive at + 10.7. Therefore, CaP nanoparticles may facilitate electrical binding to the implant’s bodily surface.

The CaP adhesion rate was as low as 11.6% when CaP was applied nonelectrically on the titanium surface in this study. Therefore, we attempted to increase the adhesion to the titanium surface by applying an electric current. When a titanium disk was immersed in the CaP suspension and an electric current was applied, the amount of CaP adhering to the titanium surface was small. The conductivity of the CaP suspension used in this study was relatively low (1832.5 μs/cm) and the current at 30 µA may have been inadequate due to this low conductivity.

It is conceivable that the applied current was insufficient and that a higher current application may increase the amount of adhered CaP. Conversely, adhesion levels were found to be greater when CaP gel was applied at the same current. Consequently, CaP nanoparticles in the gel may encounter negatively charged areas on the titanium surface generated by the applied current, leading to a stronger electrostatic adhesion to the titanium surface. The absence of significant differences in electric current application times beyond 10 min may correlate with the disappearance of charge transfer within the gel. Thus, the subsequent experiments employed a current setting of 30 µA for a duration of 5 min (at a total charge of 5.4 C). The volume of CaP adhesion exhibited a current-dependent relationship in this study. It may be feasible to achieve the same level of CaP attachment within a shorter timeframe by employing a higher current. The relationship between current magnitude and CaP adhesion volume warrants future investigation.

The Ag-purple LED treatment resulted in CaP adhesion rates comparable to those observed in the non-contamination group. Following Ag-purple LED treatment, it is plausible that the hydrophilicity of the titanium surface facilitated greater dispersal of the CaP suspension on the titanium surface. These results indicate the efficacy of Ag-purple LED treatment not only for promoting subsequent cellular attachment to the titanium surface but also for enhancing CaP coating.

No significant difference was noted in the gene transfection efficiency of the CaP nanoparticles with or without electrical energization. This finding indicates that a current of 5.4 C does not interfere with the gene transfection efficiency of CaP and that a titanium surface coated with CaP nanoparticles featuring gene transfection capabilities can be successfully generated.

The CaP coating on titanium surface induced hard tissue-forming activity of MC3T3E1 cells. Several studies have demonstrated that Ca ions enhance osteoblast differentiation and activity^42^. Notably, when integrating BMP-2 into the CaP-loaded plasmid DNA, greater calcified deposits and ALP activity were realized on the titanium surface than those produced with CaP-loaded oligonucleotide. This observation suggests that BMP-2 release from transfected MC3T3E1 cells activates the hard tissue-forming capabilities of cells and promotes calcification on the titanium surface. Tenkumo et al. previously reported that cells treated with CaP loaded with BMP-2 released BMP-2^28,29^. The removal torque values of CaP combined with plasma DNA-loaded BMP-2 were significantly higher at 14 days post-surgery, corroborating the in vitro findings. Wang et al. documented that hydroxyapatite-coated implants exhibited superior osseointegration compared to conventional titanium implants^43^. Histological examinations indicated direct bone formation on the titanium surface. The BMP-2 released from CaP-treated cells thus enhances hard tissue formation on titanium surfaces. However, the removal torque did not show improved values when CaP was loaded with oligonucleotides, likely due to insufficient quantities of CaP deposited on the titanium surface. Future research will be required to investigate conditions that further enhance CaP attachment. The osseointegration strength at 4 weeks post-surgery did not significantly differ across the groups. Cho et al. reported no notable differences in osseointegration strength when evaluating osseointegration at 1, 2, 4, 8, and 12 weeks following BMP-2 placement at the base of an implant placement hole^44^. It is plausible that the strength of osseointegration possesses an upper limit; therefore, titanium coated with CaP gene transfection carrier may serve as an effective strategy for early osseointegration.

Several experiments investigating current application to dental implants have been reported to determine the required amount of current^9,45^. However, these experiments were conducted in vitro, with current values varying significantly (22 µA–1100 mA). Furthermore, the purpose of these studies was to remove biofilms that adhered to the implant surface, which is different from that of the present study. In contrast, electroporation, a technique designed to introduce genes into cells through electric current application, typically necessitates an electric field of approximately 2000–5000 V/cm. However, given that titanium possesses an electrical resistance of 420 nΩ-m, a current ranging from 4.76 × 10⁹ to 11.9 × 10⁹ A traverses the titanium material. The electrical resistance of bone approximates 1 × 10^4^ Ω cm^46^, implying that a current magnitude of 200 to 500 mA would flow through the bone. Yoshimoto et al. documented occurrences of ventricular fibrillation at a 240 mA electric current^47^. Therefore, electroporation field intensities did not feature in this study’s design. Additionally, Nische et al. indicated that the application of 1 mA transcranial direct current stimulation (tDCS) for 60 min procured no cerebral damage^48^. An electrical root canal length measurement apparatus was utilized to ascertain the length of the root canal during dental procedures. One such device, the Root ZX (Morita Corporation, Kyoto, Japan), transmits a 30 µA current from an electrode positioned within the root canal to an electrode aligned with the buccal mucosa through several tissue types including bone, connective tissue, and muscle^49^. Given the established electrical safety, the current values employed in this study aligned with those utilized in electrical measurement devices for root canals. Future endeavors must elucidate the intricate relationships between applied current, CaP attachment levels, and gene delivery efficacy while factoring in biocompatibility.

## Limitation

*Staphylococcus aureus*, identified within peri-implantitis pockets, was employed to simulate infection of the implant surface^50^. However, a floating bacterial population or biofilm consisting of multiple bacterial species forms on titanium implant surfaces associated with peri-implantitis observed in clinical dentistry^51^. The biofilm may adhere more robustly to the titanium surface, impeding penetration of silver ions and purple LED light due to the biofilm’s inherent characteristics or thickness. Furthermore, the presence of blood and leachate from dental pockets may obstruct silver ion and light penetration. Future sterilization tests utilizing plaques obtained from peri-implantitis patients, as well as animal studies and clinical trials grounded in actual clinical practices, will be necessary.

Titanium surfaces were implanted into the femurs of rats after various treatments. Therefore, the implant surfaces did not accurately reflect those encountered in clinical settings following osseointegration loss, nor was the reacquisition of osseointegration assessed. The reacquisition of osseointegration post-peri-implantitis is influenced not only by disinfection and restoration or improvement of implant surface properties but also by multiple factors, including meticulous suturing using a gingival flap and blood supply to the gingival flap post-suturing. Given that this investigation served as a foundational experiment for clinical applications, it evaluated the effectiveness of implant surface properties with gene-delivery-capable CaP in promoting hard tissue formation and osseointegration.

The adhesive strength of CaP on titanium surfaces was not explicitly evaluated in this study. The nanoscale nature of CaP granules on the nano-roughened titanium surface precludes the application of conventional tensile testing methods. Therefore, a clinically relevant washing process was used to assess residual amounts following treatment.

The Ag-purple LED irradiation treatment used in this study offers a promising therapeutic approach with notable biological safety, as hydroxyl radicals are generated locally from bacteria through the application of visible purple LED irradiation in the presence of silver ions. Furthermore, the functionalization of CaP with gene transfer capabilities is anticipated to provide a comprehensive therapeutic strategy for peri-implantitis, facilitating the reacquisition of osseointegration.

## Conclusion

The novelty of the findings from this study is summarized as follows.

1. The combined treatment of silver ions and 400 nm purple LED irradiation, within the visible light spectrum, was found to significantly enhance bactericidal effects by generating substantial levels of hydroxyl radicals against *Staphylococcus aureus.*
2. The hydrophilicity and cell biocompatibility of the titanium surface were restored by hydroxyl radicals generated from treatments involving silver ion application on biofilm or suspension of *Staphylococcus aureus,* followed by 400 nm purple LED irradiation.
3. CaP with gene delivery capabilities was successfully deposited onto titanium surfaces electrically without compromising gene transfection efficiency at a current of 5.4 C.
4. Coating the titanium surface with CaP-associated plasmid DNA encoding BMP-2 resulted in augmented calcium deposition on the titanium surface and promoted early osseointegration.

Given the current landscape of increasing prevalence of dental implants and the associated complications arising from peri-implantitis, our study is expected to be an innovative multidimensional approach to combating biofilm infections associated with dental implants, with strong implications for improving treatment outcomes related to infection control and implant longevity. However, to facilitate the application of this comprehensive treatment in dental practice, the bactericidal effects of the combined treatment involving silver ions and 400 nm purple LED irradiation should be assessed within dental pockets in human or animal models in future research. Furthermore, optimal current application conditions should be identified to enhance CaP attachment levels and gene delivery efficacy.

## Materials and methods

### Analysis of antibacterial effect and hydroxyl radical generation on bacterial suspensions and biofilm formation on titanium surface

#### *Staphylococcus aureus* culture

In this study, *Staphylococcus aureus* JCM2413 (RIKEN BioResource Center, Wako, Japan) was utilized. *Staphylococcus aureus* was cultured on Brain Heart Infusion (BHI) agar medium (CM1136 BRAIN HEART INFUSION AGER, Kanto Chemical Co. Ltd., Tokyo) and subsequently transferred to BHI liquid medium (CM1136 BRAIN HEART INFUSION, Kanto Chemical Company, Tokyo), The culture was placed in a box containing AneroPack (Mitsubishi Gas Chemical Company, Tokyo), and incubated anaerobically for 24 h at 37 °C in an incubator. The prepared bacterial suspension was diluted to an optical density (O.D) of 1.0, using a color wave (WPA C07500, Biochrom, Cambridge, UK) using sterile saline as a diluent. This suspension contained approximately 7 × 10^8^ colony-forming units (CFUs)/mL and was used for subsequent experiments. For the biofilm-related test, 30 µl of a tenfold diluted bacterial suspension was seeded on the titanium surface and incubated in 300 µl of BHI liquid medium at 37 °C for 24 h in anaerobic conditions. Titanium disks subjected to this process were designated as the bacterial attachment group.

#### Titanium surface treatment

The titanium surface was prepared following the method described by Tenkumo et al. to mimic the SLA-treated implant surface manufactured by Straumann^18^. A commercially pure titanium disk (diameter: 5 mm; thickness: 2 mm; grade 4: TB550, Nishimura, Tokyo, Japan) was subjected to alumina sandblasting (Micro Etcher IIA, Morimura, Tokyo, Japan) for 1 min, immersed in ultrapure water, and ultrasonically cleaned for 10 min (BRANSON tabletop ultrasonic cleaner, Yamato Scientific Co., Ltd., Tokyo, Japan). Then, titanium disk was acid-etched in 48% sulfuric acid for 1 h at 60 ℃, followed by ultrasound-cleaning in ultrapure water (10 min), acetone (10 min), and finally ultrapure water (10 min). After cleaning, the specimen was autoclaved for 15 min at 121 ℃ (Autoclave SX-300, TOMY KOGYO CO., LTD., Fukushima, Japan). The sample was prepared one day before use and stored in ultrapure water. Titanium disks subjected to this procedure were designated as the non-contamination group.

#### Bactericidal assay

##### Bacterial suspension

Herein, 150 µL of the bacterial suspension (OD = 1.0) was mixed with 150 µL of various concentrations of silver nitrate solution (50, 100, 150, 200, 400, 500, 550, 600, 700 and 800 µM; 0.02 mol/L Silver nitrate solution, Wako Pure Chemical Industries, Osaka, Japan), or iron nitrate solution (75, 150, 300, 450, 600 and 800 µM; Fe 1000, Wako Pure Chemical Industries, Ltd., Osaka, Japan), or copper standard solution (75, 150, 300, 450, 600, and 800 µM; Cu 1000, Wako Pure Chemical Industries, Ltd., Osaka, Japan). Following mixing, the sample was irradiated with 400 nm purple LED light (Omnicure LX400 + ; Lumen Dynamics, Japan) at an irradiation intensity of 200 mW/cm^2^ or kept in a light-shielding box for 1 min. For subsequent reference, the combination treatment with silver, iron, or copper application and purple LED irradiation are referred to as Ag-purple LED, Fe-purple LED, or Cu-purple LED, respectively. The samples were classified into following groups based on the treatment method:

(i) Silver + LED treatment, Iron + LED treatment, Copper + LED treatment: mixing silver solution, iron solution, or copper solution with bacterial suspension followed by purple-LED irradiation;
(ii) Silver-No LED treatment, Iron-No LED treatment, Copper-No LED treatment: mixing silver solution, iron solution, or copper standard solution with bacterial suspension followed by incubation in a light-shielding box;
(iii) Control (Water)-No LED: mixing ultrapure water with bacterial suspension followed by incubation in a light-shielding box.

A 100-µl aliquot of the prepared reaction solution was added to 100 µl of Mueller–Hinton Broth (MHB; 370 mg/ml: CM0405B, Kanto Chemical Co., Inc., Tokyo) to halt the antibacterial activity of the residual silver, iron and copper ions. Next, the treated solution was diluted 10–10^5^-fold with saline, after which 10 µL of the diluted solution was seeded on BHI agar to evaluate the treatment effect. The agar plates were anaerobically cultured at 37 ℃ for 24 h followed by colony enumeration for CFU/mL determination.

##### Biofilm on the titanium surface

The biofilm on the titanium surface was prepared as aforementioned. After removing the culture solution from the prepared biofilm, the titanium was washed with phosphate-buffered saline (PBS) twice. After removing PBS, the titanium disks with biofilm were immersed in 300 µl of silver nitrate solution (final concentrations: 50, 100, 150, 200, 400, 500, 550, 600, 700, or 800 µM). Subsequently, the cells were exposed to purple-LED (400 nm) irradiation or kept in a dark room for 1 min. The treated titanium plates were washed with Milli-Q (MQ) water and incubated in 300 µL of type I collagenase solution containing Disperse, agitating 37 °C for 2 h to extract remaining bacteria from the titanium surface. The supernatant was collected and the solution was further diluted 10–10^5^-fold with saline, and subsequently 10 µL of the diluted solution was seeded on BHI agar to determine the treatment effect. The agar plates were anaerobically cultured at 37 ℃ for 24 h followed by colony enumeration to determine CFU/mL. The samples were classified into the following groups depending on the treatment methods:

(i) non-contamination: titanium disk subjected solely to alumina sandblasting, acid-etching, and ultrasound-cleaning without biofilm formation;
(ii) biofilm: titanium disk subjected solely to alumina sandblasting, acid-etching, and ultrasound-cleaning, followed by biofilm formation;
(iii) Silver + LED treatment: titanium disk with biofilm immersed in silver solution, followed by purple-LED irradiation; and
(iv) Silver-No LED treatment: titanium disk with biofilm immersed in silver solution, followed by incubation in a light-shielding box.

#### Analysis of hydroxyl radical generation

##### Bacterial suspension

For this analysis, 75 µL of silver nitrate, iron nitrate, or copper nitrate solution (final concentrations: 0, 75, 150, 300, 450, 600, 800 µM) or ultrapure water were mixed with 50 µL of 5,5-dimethyl-1-pyrroline-N-oxide (DMPO; FUJIFILM Wako Pure Chemical, Osaka), followed by the addition of 75 µL of the bacterial suspension (OD = 1.0). The prepared solution was irradiated with a purple LED light (400 nm) or incubated in a light-shielding box for 1 min. The samples were classified into following groups based on the treatment method:

(i) S.a-Ag: siver solution mixed with bacterial suspension of *Staphylococcus aureus* in a light-shielding box for 1 min;
(ii) S.a-Ag-LED: purple-LED irradiating the mixture of silver solution and bacterial suspension of *Staphylococcus aureus* for 1 min;
(iii) S.a-Fe: iron solution mixed with bacterial suspension in a light-shielding box for 1 min;
(iv) S.a-Fe-LED: purple-LED irradiating the mixture of iron solution and bacterial suspension of *Staphylococcus aureus* for 1 min;
(v) S.a-Cu: copper solution mixed with bacterial suspension of *Staphylococcus aureus* in a light-shielding box for 1 min;
(vi) S.a-Cu-LED: purple-LED irradiating the mixture of copper solution and bacterial suspension of *Staphylococcus aureus* for 1 min; and
(vii) S.a-LED: purple-LED irradiating the mixture of ultrapure water and bacterial suspension of *Staphylococcus aureus* for 1 min.

Each reacted solution was analyzed using an X-band electron spin resonance (ESR) spectrometer (JES-FA-100, JEOL, Tokyo, Japan) and the ESR spectrum of each sample was recorded. The concentration of number of radicals generated (DMPO-OH) was determined using Digital Data Processing software (JEOL). The tests were performed in triplicates.

##### Biofilm

A biofilm was prepared on the titanium surface, as described above. The culture solution of the prepared biofilm was removed, and the titanium was washed twice with PBS. Following PBS removal, titanium disks with biofilm were immersed in a mixture of 75 µL of silver nitrate (final concentration: 600 µM) or 75 µL of ultrapure water, and 50 µL of DMPO. The mixture was either irradiated with purple-LED or incubated in a light-shielding box for 1 min. As a control, titanium without biofilm, prepared according to the method in Sect. 4.1.2, was immersed in silver nitrate solution and subsequently exposed to purple LED light. The ESR spectra were recorded for each sample as described above, and the concentration of DMPO-OH was quantified.

#### Cytotoxicity assessment

MC3T3E1 osteoblastic cells (RIKEN CELL BANK, Tsukuba, Japan) were cultured in Dulbecco’s Modified Eagle Medium (DMEM, SIGMA, Japan) supplemented with 10% fetal bovine serum (FBS, Thermo Fisher Scientific, USA) and 1% penicillin/streptomycin (Wako Pure Chemical Industries, Osaka, Japan) at 37 °C in a 5% CO_2_ environment. MC3T3E1 cells were seeded in 96-well plates at a density of 3 × 10^3^ cells per well and incubated overnight at 37 °C in a 5% CO_2_ atmosphere. The culture medium was removed and 100 μL of silver nitrate solution (1600 μM) was added to the wells, which were irradiated with purple-LED light for 1 min. Cell viability was assessed using the 3-(4,5-dimethylthiazol-2-yl)-2,5-diphenyltetrazoliumbromide (MTT; Sigma, Japan) assay according to our previously reported protocol^18^. The culture medium was replaced with MTT solution (final concentration 1 mg/mL), followed by incubation for 1 h at 37 °C under a 5% CO_2_ atmosphere. The MTT solution was subsequently replaced with DMSO, followed by incubation for 30 min at room temperature. Absorbance of the reacted solution was measured at 570 nm using a microplate reader (Spectra MAX 190; Molecular Devices, Japan). As a control, untreated MC3T3E1 cells at a density of 3 × 10^3^ cells per well were subjected to silver nitrate (1600 μM) in a light-shielded box for 1 min or PBS in purple-LED irradiation for 1 min. The reacted solution was removed and 100 μL of MTT solution was added, adhering to the same protocol. Absorbance of the cells was normalized to that of the control. The experimental groups were classified into following groups based on the treatment method:

(i) Mock: PBS application in a light-shielding box;
(ii) Silver alone group: Silver nitrate solution application in a light-shielding box;
(iii) LED alone group: PBS application followed by purple-LED irradiation; and
(iv) Silver + LED group: Silver nitrate solution application followed by purple-LED irradiation.

#### Examination of titanium surface properties

##### Electrical properties

The titanium surface with a microstructure was treated as described above. The surface charge of titanium without bacteria was measured from a distance of 5 mm using a digital low-voltage measuring instrument (MODEL KSD-3000, Kasuga Electric Co., Ltd., Kawasaki, Japan), while applying a current of 0 or 10 mA and a voltage of 0.5 V (Direct Current Stabilized Power Supply AD-8724D; A&D Company, Ltd., Tokyo) to the titanium surface.

##### Hydrophilic properties

Ultrapure water (2 µl) was deposited onto the titanium surfaces of the non-contamination group, bacterial adhesion group, Silver-No LED treatment group, or Silver + LED treatment group, which were prepared as described above. The final concentration of silver ion was 1600 µM. Contact angles were measured (Contact Angle Meter CA-X, Kyowa Interface Science Co., Saitama, Japan).

##### Scanning Electron Microscope (SEM) assessment

The titanium surfaces of the non-contamination group, biofilm group, Silver-No LED treatment group, or Silver + LED treatment group were observed using a scanning electron microscope (SEM, SU-5000, Hitachi, Japan). The final silver ion concentration was 1600 µM.

#### Cell viability assessment

MC3T3E1 cells were seeded at a density of 3.0 × 10^4^ on the titanium surfaces of the non-contamination group, Silver-No LED treatment or Silver + LED treatment groups, and subsequently cultured at 37 °C in a 5% CO_2_ environment. After 3 days, cell viability was assessed using the MTT assay as previously described. The non-contamination group served as the standard.

### CaP coating on titanium surface

#### Preparation of CaP

CaP nanoparticles were prepared according to a previously reported method^29^. Briefly, 18 mM calcium nitrate tetrahydrate and 10.8 mM diammonium hydrogen phosphate were adjusted to pH 9.0 using hydrochloric acid and sodium hydroxide. Equal volumes of each solution were mixed using a peristaltic pump. Next, 100 µl of the reacted solution was supplemented with 40 µl of oligonucleotide (1 mg/ml; Poly(I:C), Funakoshi Corporation, Tokyo, Japan), plasmid-DNA loading mCherry (1 mg/ml; mCherry_pcDNA3.1(+)-C-HA, GenScript, Tokyo, Japan), or BMP-2 (1 mg/ml; BMP-2_pcDNA3.1(+)-C-HA, GenScript, Tokyo, Japan). Subsequently, 50 µl of calcium nitrate tetrahydrate and 50 µl of diammonium hydrogen phosphate were added to the prepared mixture solution, followed by the addition of 40 µl of protamine (1 mg/ml; Wako Pure Chemical Industries, Ltd., Japan), which was thoroughly mixed. The prepared solution was centrifuged (1.0 × 10^4^ rpm) for 5 min. The supernatant was discarded and the remaining precipitate was used in subsequent experiments as CaP. Based on the oligonucleotide or plasmid DNA contained, they were designated as CaP (oligo), CaP (mCherry), and CaP (BMP-2). The CaP (oligo) was observed under an SEM. The zeta potential and particle size were determined using a Zetasizer nanoseries instrument (ELSZ-2, Otsuka Electronics, Osaka, Japan). The conductivity of CaP (oligo) was measured using an electrical conductivity meter (LAQUAtwin-EC-33B; HORIBA, Kyoto, Japan).

#### Calcium concentration

The calcium concentration of the extracted solution in each session was measured using inductively coupled plasma optical emission spectrometry (ICP-OES), and the concentration of calcium phosphate in the CaP was calculated. The average value was obtained from three independent measurements.

#### CaP nanoparticle coating

After CaP (oligo) was diffused into 280 µl of sterile water to prepare a CaP suspension, the titanium from the non-contamination group was immersed in the CaP suspension. The 280 µl of CaP suspension contained 3.24 µg of CaP nanoparticles. Groups were defined based on the coating methods as follows:

(i) Immersion + 30 µA group: an anode terminal connected to the titanium disk with a cathode terminal placed in a CaP suspension of the potentiostat/galvanostat (Hokuto Denko Co., Ltd., Tokyo, Japan), and a current of 30 µA flowed for 5 min;
(ii) Immersion − 30 µA group: a cathode terminal connected to the titanium disk with an anode terminal placed in a CaP suspension of the potentiostat/galvanostat, and a current of 30 µA flowed for 5 min; and
(iii) Addition 30 µA groups: the precipitated CaP (oligo) was applied to the titanium disk, with an anode and a cathode terminal of the potentiostat/galvanostat connected directly to the titanium disk, and a current of 30 µA applied for 5 min.

After rinsing with sterile water, the titanium discs were immersed in 20 mL of 1 M nitric acid at 4 °C for 3 days to elute calcium. The calcium concentration was measured using ICP-OES.

#### Scanning Electron Microscope

Non-contaminated titanium disks and Silver + LED treatment groups were prepared. CaP(oligo) was seeded on the disk, and the anode and cathode terminals of the potentiostat/galvanostat were directly connected to the titanium disk, and a current of 30 µA was applied for 5 min. The surface of the titanium disk was rinsed with sterile water and observed under an SEM(SU-5000, Hitachi, Japan).

#### CaP nanoparticle coating condition

##### Current value

CaP (oligo) was applied to the non-contaminated titanium, with an anode and cathode terminal of the potentiostat/galvanostat connected directly, and a current of 0, 15, or 30 µA (current at 0, 4.5, or 9 C) applied for 5 min. After rinsing with sterile water, the titanium discs were immersed in 20 mL of 1 M nitric acid at 4 °C for 3 days to elute calcium. The calcium concentration was measured using ICP-OES.

##### Energization time

CaP (oligo) was applied to the non-contaminated titanium, with an anode and a cathode terminal of the potentiostat/galvanostat connected directly, and a current of 30 µA (current level: 9 C) was applied for 1, 3, 5 and 10 min (current levels: 1.8, 5.4, 9.0 and 18.0 C). After rinsing with sterile water, the titanium discs were immersed in 20 mL of 1 M nitric acid at 4 °C for 3 days to elute calcium. The calcium concentration was measured using ICP-OES.

##### Ag-purple LED treatment

CaP (oligo) was applied to the titanium surfaces of the non-contamination, bacteria-attached, or Silver + LED treatment groups, with an anode and a cathode terminal of the potentiostat/galvanostat connected directly, and a current of 30 µA was applied for 5 min (as in the Addition 30 µA group). After rinsing with sterile water, the titanium discs were immersed in 20 mL of 1 M nitric acid at 4 °C for 3 days to elute calcium. The calcium concentration was measured using ICP-OES.

##### Gene transfection efficiency

To determine the effect of electronic stimulation on gene transfection, the following groups were established:

(i) CaP(mCherry/E+) group: CaP(mCherry) gels seeded on titanium surfaces of the non-contamination group, with an anode and a cathode terminal of the potentiostat/galvanostat connected directly, and a current of 30 µA applied for 5 min (as in the Addition 30 µA group);
(ii) CaP(mCherry/E−) group: CaP(mCherry) gels seeded on the titanium surfaces of the non-contamination group and left for 5 min without current application.

Subsequently, MC3T3E1 cells were seeded at 5.0 × 10^4^ cells per titanium disk and cultured for 3 days at 37 °C in a 5% CO_2_ environment. The volume of applied Plasmid-DNA was 40 µg. The samples were fixed in a 4% formalin solution for 10 minutes and washed twice with PBS. The samples were stained with 300 µL of DAPI(4',6-diamidino-2-phenylindole, dihydrochloride; Thermo Fisher Scientific, Japan) for 5 minutes, and washed twice with PBS. Gene transfer efficiency was observed using a fluorescence microscope (Digital Imaging System (APX100), EVIDENT, Tokyo). Gene transfection efficiency was calculated as fluorescent cell/DAPI stained cells.

##### Biocompatibility of CaP coating

To evaluate the ability of the CaP coatings to sustain cell viability under varying titanium conditions, the following groups were established:

(i) Non-contamination group: 3.0 × 10^4^ of MC3T3E1 cells were seeded on non-contaminated titanium discs;
(ii) Silver + LED treatment group: 3.0 × 10^4^ of MC3T3E1 cells were seeded on titanium surfaces treated with Silver + LED;
(iii) CaP(oligo/E +) group: CaP (oligo) was applied to titanium treated with Silver + LED, followed by a 30 µA current application for 5 min (as in the Addition 30 µA group).

Finally, MC3T3E1 cells (3.0 × 10^4^) were seeded on the titanium disks. After cell seeding, all groups were cultured at 37 °C in a 5% CO_2_ atmosphere. The MTT assay was performed 3 days later.

##### Bone formation activity and calcification

CaP (oligo) and CaP (BMP-2) were prepared as described previously. Both CaP nanoparticles were placed on the titanium surfaces in the non-contamination and Silver + LED treatment groups. Thereafter, 30 µA of current was directly connected to the titanium disk for 5 min (as in the Addition 30 µA group). MC3T3E1 cells (3.0 × 10^4^) were seeded onto the prepared titanium disk and cultured in Dulbecco’s modified eagle medium containing 10% FBS and 1% penicillin streptomycin for 30 days at 37 °C and 5% CO_2_. The culture medium was collected at 3, 6, 9, 12, 16, 19, 23, 26, and 30 days after cell seeding and replaced with fresh medium. Groups were defined based on the treatment methods as follows:

(i) Non-contamination group: MC3T3E1 cells seeded on non-contaminated titanium screws;
(ii) Silver + LED group: MC3T3E1 cells seeded on titanium surfaces treated with A(+)L(+);
(iii) CaP (oligo/E+) group: CaP(oligo) applied to the Silver + LED treated titanium surface, with 30 µA current connected directly to the titanium screw for 5 min (as in the Addition 30 µA group), and MC3T3E1 cell were seeded; and
(iv) CaP (BMP-2/E+) group: CaP(BMP-2) applied to the Silver + LED treated titanium surface, with 30 µA current connected directly to the titanium screw for 5 min (as in the Addition 30 µA group), and MC3T3E1 cell were seeded.

The alkaline phosphatase (ALP) activity of the collected culture medium on days 3, 6, 9, 12, 16, 19, 23, 26 and 30 was measured using an ALP laboratory assay (Fujifilm Wako Pure Chemicals Co., Ltd., Osaka, Japan) in accordance with the manufacturer’s protocol. Absorbance was analyzed at 415 nm using absorbance spectrophotometry (Spectra MAX 190, Molecular Devices, Japan). The ALP activity values for each measurement day were accumulated to calculate the total ALP activity after 30 days.

In contrast, calcification was evaluated as follows. After 30 days, the cells were fixed in methanol and stained with alizarin red. The dye was eluted with 5% formic acid, and the absorbance was analyzed at 415 nm using absorbance spectrophotometry.

### Osseointegration of titanium screw

#### Preparation of titanium screw

CaP (oligo) or CaP (BMP-2) gel (140 µl), each containing 40 µg of plasmid-DNA, were prepared as descried above. A titanium screw was designed to mimic an SLA-treated implant surface manufactured by Straumann. Briefly, a commercially pure titanium screw (diameter 1.6 mm, length 3 mm; FUKUOKA SEIMITU, Japan) was subjected to alumina sandblasting for 1 min, followed by acid-etching in 48% sulfuric acid for 1 h at 60 ℃ and subsequently cleaned by ultrasonic treatment in ultrapure water and acetone. The screw was finally autoclaved for 15 min at 121 ℃. Following this, 30 µl of a tenfold dilution of *Staphylococcus aureus* suspension (OD = 1.0) was seeded for each titanium screw. Next, 300 µl of BHI liquid medium was added and incubated under anaerobic conditions for 48 h to prepare a biofilm on the implant screw, simulating titanium affected by peri-implantitis. After silver + LED treatment, the prepared CaP (oligo) or CaP (BMP-2) gel was applied, and 30 µA of current was directly applied for 5 min. The groups were defined based on the plasmid DNA content as follows: CaP(oligo/ E +) and CaP(BMP-2/E +). As a control group, the following groups were established: non-contamination group (non-contaminated titanium screw) and Silver + LED treatment group (titanium screw receiving Silver + LED treatment after biofilm formation on titanium surface). A total of four groups were prepared.

##### Animal care and management

The rats were housed in plastic cages with dimensions of height: 403 mm, width: 345 mm, and length: 317 mm. Each cage accommodated 2–3 rats. Fresh water (Edstrom industries INC, Wisconsin, USA) was provided automatically and Labo MR Stock (Nosan, Kanagawa, Japan) was used as their food, with the quantity checked weekly. Aspen chips (CL-4169, CLEA, Japan) were used as bedding, and both the bedding and cages were changed every week. A brown transparent plastic house was established, designed to accommodate up to five mice simultaneously. The lights in the bleeding room were switched off at 20:00 and turned on at 08:00. The air conditioning was automatically controlled using a clean air system (Clea, Kanagawa, Japan).

##### Implant placement

In this experiment, 20 8-week-old Wistar rats (7 weeks old; 200–230 g; male) were purchased from CLEA Japan, Inc (Japan), and used in accordance with the guidelines for the care and use of laboratory animals at Tohoku University (2023DnA-001-01). The rats were anesthetized by inhalation with isoflurane (Fujifilm Wako Pure Chemicals, Osaka, Japan) and intraperitoneally with a triad of medetomidine (Domitor, 0.15 mg/kg, Nippon Zenyaku Kogyo, Japan), midazolam (Sandoz, 2 mg/kg, Sandoz, Japan), and butorphanol (2.5 mg/kg, Meiji Seika Co., Japan). After shaving the thigh, a skin incision was made and the muscle was detached to expose the femur. A bone defect (1.68 mm diameter) was created using a 1.68 mm diameter round bur (GC, Japan). The titanium screws were then implanted into the bone defects. Ten implants were allocated to each group, with one implanted randomly in each foot. After implantation, the muscles and skin were tightly sutured to preventcontamination, exposure, or detachment of the titanium screw. At 14 and 28 days after implantation, the rats were anesthetized by inhalation of isoflurane and intraperitoneally administered a triad of anesthetics (medetomidine, midazolam, and butorphanol). The removal torque test of the implanted titanium screw was performed by rotating the Dezirache (No. GLK060; KTC, Tokyo, Japan) in reverse. The rats were euthanized using an overdose of sodium pentobarbital. Additionally, the tissues surrounding the non-contaminated and CaP(BMP-2, E+) groups were extracted and fixed in 4% formaldehyde phosphate for 2 days. Non-decalcified specimens were prepared and stained with hematoxylin and eosin (H&E) stain for histological observation. All animal experimental protocols were reviewed and approved by the Institutional Animal Experiment Committee of Tohoku University prior to the commencement of the animal experiments.

### Statistical analysis

We used JMP® Pro 17.0.0(https://www.jmp.com) for statistical analysis. All data are presented as means ± standard deviation (SD). Normal distribution of the data was verified using the Shapiro–Wilk test. For p-values > 0.05, a t-test was performed between the two groups, and a Tukey–Kramer test was performed for comparisons among more than two groups. When the p-value was < 0.05, the Wilcoxon test was utilized for two groups, and the Steel–Dwass test was applied for comparisons involving more than two groups.

## Data Availability

The data supporting the findings of this study are available from the corresponding author upon reasonable request.
